# A Machine Learning‐Based Approach to Clinopyroxene Thermobarometry: Model Optimization and Distribution for Use in Earth Sciences

**DOI:** 10.1029/2021JB022904

**Published:** 2022-04-09

**Authors:** C. Jorgenson, O. Higgins, M. Petrelli, F. Bégué, L. Caricchi

**Affiliations:** ^1^ Department of Earth Sciences University of Geneva Geneva Switzerland; ^2^ Department of Physics and Geology University of Perugia Perugia Italy

**Keywords:** machine learning random forest, clinopyroxene thermobarometry, model optimization

## Abstract

Thermobarometry is a fundamental tool to quantitatively interrogate magma plumbing systems and broaden our appreciation of volcanic processes. Developments in random forest‐based machine learning lend themselves to a data‐driven approach to clinopyroxene thermobarometry, allowing users to access large experimental data sets that can be tailored to individual applications in Earth Sciences. We present a methodological assessment of random forest thermobarometry using the R freeware package extraTrees. We investigate the model performance, the effect of hyperparameter tuning, and assess different methods for calculating uncertainties. Deviating from the default hyperparameters used in the extraTrees package results in little difference in overall model performance (<0.2 kbar and <3°C difference in standard error estimate, SEE). However, accuracy is greatly affected by how the final value from the distribution of trees in the random forest is selected (mean, median, or mode). Using the mean value leads to higher residuals between experimental and predicted P and T, whereas using median values produces smaller residuals. Additionally, this work provides two scripts for users to apply the methodology to natural data sets. The first script permits modification and filtering of the model calibration data set. The second script contains premade models, where users can rapidly input their data to recover PT estimates (SEE clinopyroxene‐only model: 3.2 kbar, 72.5°C and liquid‐clinopyroxene model: 2.7 kbar, 44.9°C). Additionally, the scripts allow the user to estimate the uncertainty for each analysis, which in some cases is significantly smaller than the reported SEE. These scripts are open source and can be accessed at https://github.com/corinjorgenson/RandomForest-cpx-thermobarometer.

## Key Points

- Random forest machine learning is a novel approach for clinopyroxene thermobarometry with a comparable error to thermodynamic thermobarometers
- Model optimization was performed, and model performance is insensitive to most hyperparameters
- A detailed description of how to access and use the model is included so readers can use the thermobarometer

## 1 Introduction

Quantifying the pressure and temperature of mineral crystallization is an invaluable method to retrieve information on architecture of volcanic plumbing system of volcanoes and constrain magma migration and storage through the lithosphere (Giacomoni et al., 2016; Ridolfi et al., 2008; Shane & Smith, 2013; Shaw, 2018a; Smith, 2013). Clinopyroxene chemistry has been widely used for this endeavor by calibrating thermobarometers (Masotta et al., 2013; Neave & Putirka, 2017; Putirka, 2008; Wang et al., 2021). Classically, these thermobarometers result in a single equation, which links site‐specific mineral chemistry (plus or minus equilibrium liquid data) to the variation in pressure or temperature of crystallization. However, these formulas are often associated with large standard error estimates (SEE) and are only appropriate for specific melt compositions (e.g., Neave & Putirka, 2017 for ultramafic to intermediate compositions; Masotta et al. (2013) for alkaline magmas). Additionally, early thermobarometer calibrations were self‐validated, which means that data used to regress the model are also used to validate it. This typically leads to data overfitting and an underestimated SEE (Nimis & Taylor, 2000; Putirka, 2008). Recent developments in machine learning applications to petrology by Petrelli et al. (2020) and Higgins et al. (2022) have resulted in a machine learning random forest approach to thermobarometry. Both studies omitted several pertinent experimental data sets of clinopyroxene and liquid equilibria, which are now included in the model presented here.

**Figure 1:**
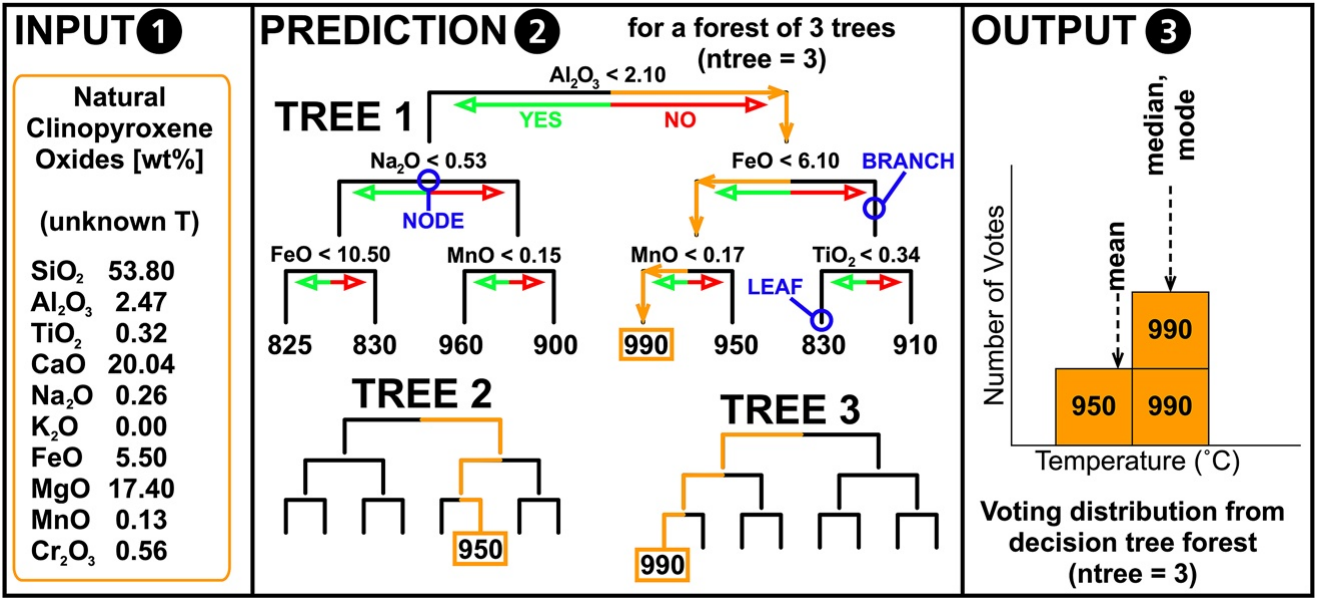
Process of determining temperature from a natural (unknown T) clinopyroxene using machine learning thermobarometry. The input to the model (1) is the chemistry of the natural clinopyroxene. The chemical composition is cascaded through each decision tree in turn (2; orange path), arriving at the temperature at the base of each tree. The voting distribution (3; output) is used to determine the temperature. This temperature can be selected based on the mean, median or mode of the voting distribution (see text for details).

Random forest is a machine learning method that employs decision trees to populate an improved prediction‐based model, using the results from a distribution of hundreds of trees to generate an output (Breiman, 2001, 2002; Ho, 1995). A decision tree is a hierarchical flowchart that determines an outcome when given a set of input variables (Figure 1). Each tree is composed of branches and leaves, where the branches represent different pathways from the root to the desired outcome (the leaves). Branches split at nodes, where at each node, a branch may spilt either left or right in the simplest case. When a branch can no longer split, a leaf is “grown,” and the desired output is reported. In our case, the branches and nodes are dictated by clinopyroxene geochemistry, and the leaves are pressure (*P*) or temperature (*T*) of crystallization. However, the chemical element (or oxide) selected at each node greatly influences the predictive outcome of the tree. Hence, the random forest model is composed of hundreds of decision trees. Therefore, from these hundreds of decision trees, the output (predicted *P* or *T*) is the mean value from all decision trees in the case of regressive models. To allow the model to construct reasonable decision trees for prediction of natural data, we input a data set of experimentally derived clinopyroxenes (e.g., Figure S1 in Supporting Information S1) with a known pressure and temperature of crystallization, hereafter referred to as the calibration data set. In principle, the idea is very simple—the algorithm uses the calibration data set to create a predictive model, which we can apply to natural samples. However, there are several parameters to consider when calibrating a model for reliable prediction of natural data, in addition to several statistical metrics for selecting the best estimation from the voting distribution of decision trees (e.g., mean, median, or mode). Importantly, the performance of the algorithm is assessed using experiments that were not included in the calibration data set. This is done by extracting a testing data set from the initial data set that does not see the model prior to testing. This process is repeated 200 times for statistical significance and allows all the data of the data set to be used without using self‐validating methods.

Increasingly, models and methodologies for Earth science applications have moved to powerful and adaptable codes for programs such as R, python, and MATLAB as well as hosted on online servers, such as github (Georgeais et al., 2021; Ghiorso & Wolf, 2019; Iacovino et al., 2020; Lemenkova, 2019; Lubbers et al., 2019). This allows for more user interaction and in some cases provides open‐source options to users regardless of their operating system or access to apps like excel. Thus, the twofold aim of this work is to (a) build and test the performance of a thermobarometer model for clinopyroxenes and (b) provide a comprehensive explanation of how to apply our thermobarometer for applications to natural data. Our regression strategy offers a generalized model that can be tailored for certain settings, applications, or other suitable mineral phases (e.g., amphibole; Higgins et al., 2022). We greatly expand the data set of clinopyroxene and liquid equilibria in our calibration data set compared to previous studies, allowing our calibrations to be as globally applicable and adaptable as possible for users.

## 2 Methods

### 2.1 Data Sets and Preprocessing

The calibration data set is composed of experimentally grown clinopyroxenes and equilibrium liquids compiled from the Library of Experimental Petrology Research database and additional works not included in the LEPR database (Hirschmann et al. (2008); Table S1 in Supporting Information S1). The unfiltered calibration data set features 2571 datapoints, including temperatures from 679 to 2180°C, 0–160 kbar, and 6.5–78.18 wt.% SiO_2_. Following the works of previous thermobarometers, we use an equilibrium filter on the basis of the Fe‐Mg exchange, accepting only data within a 1 sd of the average Kd_Fe‐Mg_ of the unfiltered data set (Klügel & Klein, 2006; Putirka, 2008, 2016; Ziberna et al., 2016; Figure S1a in Supporting Information S1). This is a relatively stringent test but may be adapted by users within the script if preferred. The data were then filtered to remove the rare high‐pressure experiments (>30 kbar) or low SiO_2_ liquid contents (<35 wt. % SiO_2_). These regions are removed as they are rare in the calibration data set and we find poorer performance from the model for regions of the calibration data set with sparse data due to the limited extrapolative ability of machine learning. This forms the final calibration data set (*n* = 2079, Table S1, Figure S1 in Supporting Information S1). The role of H_2_O in the melt may also be an indicator of PT conditions, but as it is not consistently reported in the literature, we omit it from these models (Behrens et al., 2009; Ghiorso & Gualda, 2015; Newman & Lowenstern, 2002).

Typically, thermobarometers are calibrated and tested in the following way. First, a large (>80% of total experiments) training data set is selected from the total calibration data set of experiments. This data set is used to calibrate with the chosen regression strategy (e.g., linear regression and multivariate linear regression). The remaining data are placed into a test data set, which is used to assess the performance of the model. This is commonly achieved by running each composition in the test data set through the regressed model and calculating the standard error estimate or distribution of residual values to the known experimental values (Putirka, 1999, 2008; Ridolfi et al., 2008).

The pressure‐temperature distribution of the calibration data set is not uniform—experiments are preferentially run at low pressures. Thus, randomly extracting from the calibration data set unevenly weights the test set to have low pressure experiments, resulting in a poor representation of the SEE. To circumvent this issue, our test data set was uniformly extracted from the calibration data set on a gridded basis (Figure S1b in Supporting Information S1). Sampling from a gridded distribution offers additional biases, such as oversampling PT grid spaces, that may have a small distribution of data (i.e., a grid may have only 1 out of the total 2079 points sampled)—thus, the grid spacing was randomized for each 200 runs and samples were not extracted if the grid space did not have at least two datapoints. This results in each test data set sampling approximately a tenth of the total calibration data set. Once the respective test and train data sets are extracted, then the model is run for each set (200 times). By generating multiple random splits of test and train data sets, we can evaluate the full effect of sampling on the SEE (and other model performance metrics). This effect is not considered in conventional calibration methods (e.g., Ridolfi et al., 2010; Ridolfi & Renzulli, 2012). This methodology has benefits over a weighted mean as it removes data with an inverse proportionality to the number of experiments performed at specific conditions, while not removing single experiments performed within a single element of our grid. We note that the calibration data set has pressure and temperature uncertainties associated with different experimental setups, for example, temperature gradients along a capsule. These errors, however, are sufficiently small when compared to the calibration SEE and thus are not propagated through the model. Finally, experiments performed at the highest pressures are also performed at the highest temperatures, which make experimental pressure and temperature not independent. This aspect should be considered especially when temperature is used as an input parameter to retrieve pressure information as it could lead to biased pressure estimates. In our model, we do not use temperature as an input parameter to estimate pressure.

### 2.2 Components of a Random Forest

We chose to use the package extraTrees developed by Simm et al. (2014) although the randomForest package by (Breiman, 2002) produces comparable results at greater computational expense (Petrelli et al., 2020). The extraTrees package includes several parameters that can affect model performance. First, ntree (default = 500) determines the number of individual decision trees, which are used for prediction. A sufficiently high number of trees must be used to provide stability of the variable importance. The number of trees should be considered a convergent variable, where beyond a certain threshold performance improvement is marginal and the number of trees should be minimized to save on computational time without sacrificing performance (Breiman, 2001; Probst & Boulesteix, 2018; Probst et al., 2019; Sage et al., 2020). Second, mtry dictates how many variables (in our case, the major element chemical constituents of clinopyroxene) are considered at each node. The mtry is more influential on the overall performance of the model and default mtry for extraTrees is the total number of variables divided by three (Probst et al., 2019; Simm et al., 2014). For each node in a decision tree, a random subset of variables equal to mtry are selected from which the best performing variable is eventually chosen. In extraTrees, each node is split at a random value as described (Simm et al., 2014). To choose which of the selected variables is used for the next node, a score is calculated for each variable for regressive models. This score is calculated considering a proportional negative variance for each split (denoted by *L* for left and *R* for right).

(1) $$score=n_{L}*var_{L}+n_{R}*var_{R}$$

(2) $$var=-\frac{1}{n}\overset{n}{\underset{i=1}{\sum }}{\left(y_{i}-mean\left(y\right)\right)}^{2}$$

where *n*_L_ and *n*_R_ are the number of datapoints assigned to each left or right branch, and var is the negative variance of the data on the left (or right) side of the split for the *y* variables (Simm et al., 2014). The tested variable with the highest score is chosen for the node (See Figure S2 in Supporting Information S1 for further explanation).

The extraTrees package provides an additional variable for modification, which is the number of random cuts (numRandomCuts; the number of branches at a given node) where greater than two splits is termed nonbinary splitting. As noted in the extraTrees vignette, optimization may occur when using numRandomCuts between 3 and 5. We found a minor improvement in the SEE (<0.02 kbar), but the increase in computational time negated any positive effects of more splits.

Each decision tree generates a single output value and thus a forest with 300 trees generates 300 pressure or temperature estimates. By default, in regression mode, random forest algorithms consider the mean or the mode values to return an estimate in regression or classification mode, respectively. In addition to the mean, we additionally calculate the median and modal estimates to evaluate the model performance. The median is calculated by taking the middle value from a sorted set of values. Thus, to avoid the rare case where there is an even number of trees, and the two center points are drastically different, we have decided to use an odd number of trees to average the two values.

### 2.3 Error Assessment

Before continuing, we must consider the argument of accuracy versus precision. Random forest is effective at generating precise values, but a reliable thermobarometer needs to be accurate as well as precise. As such, the evaluation of the uncertainty of an individual model based on R^2^ values (Equation 3, where RSS is the residual sum of squares and TSS is the total sum of squares) and the residual values (absolute difference between the experimental temperature or pressure and the temperature or pressure output from the model), in addition to the standard error estimate (SEE) and the interquartile range (IQR) of the voting distribution.

(3) $$R^{2}=1-\frac{RSS}{TSS}$$

To avoid self‐validation and overfitting, data within the test data set must not be used in the data set, which trains the model (training data set). Varying the test data set is one of the largest sources of variation in the SEE and so we resampled the calibration data set 200 times to produce 200 separate tests and training data sets. Then, the average SEE is taken from the distribution of errors for all 200 data set splits. Two hundred runs were chosen as this is the minimum number of runs where the SEEs are normally distributed, thus preserving computational cost while maintaining a representative assessment. When the chemistry of the mineral under consideration is close to one of the analyses from the experiments, the distribution of estimates is characterized by low values of the IQR (which can be significantly smaller than the SEE). Therefore, we also use the IQR to calculate a confidence interval of each estimated value. We recommend users remove natural data estimates for which the IQR is double the models SEE as these are considered outside of the model error (e.g., for a model SEE of 3.4 kbar use an IQR filter of 6.8).

## 3 Results

### 3.1 Hyperparameter Tuning

Hyperparameter tuning aims to structure the best performing model possible (Breiman, 2002; Probst & Boulesteix, 2018). To systematically test the effect of hyperparameter variability, we ran 19,980 simulations, which encompass 90 combinations ranging from 1 to 9 mtry and 101–1001 ntrees where each permutation is run 200 times with the respective test and train data sets to determine the average SEE and R^2^, calculated using the ideal median pressures and temperatures.

**Figure 2:**
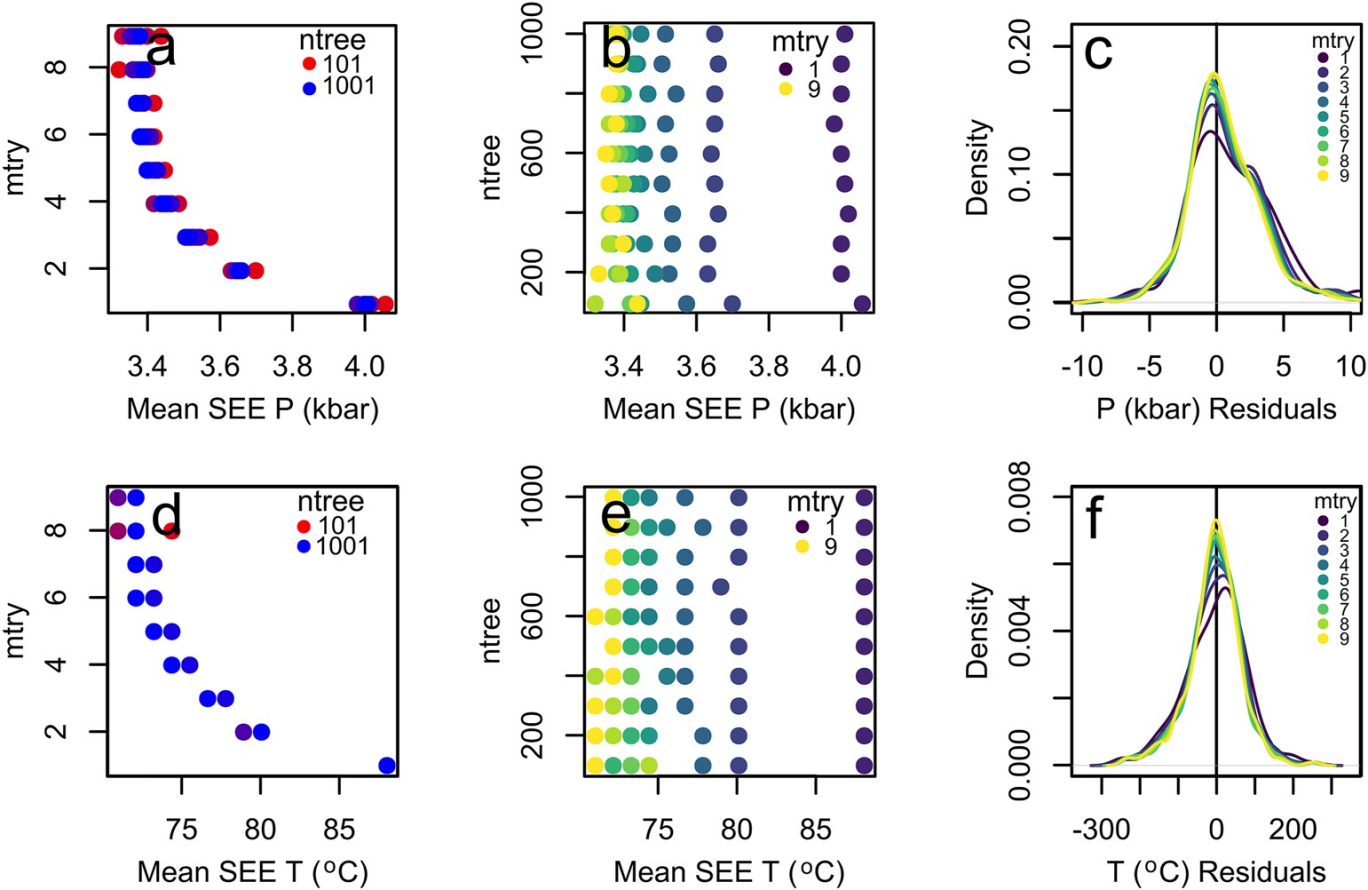
Distribution of the mtry (a and d), ntree (b and e), and residuals (c and f) for both pressure and temperatures calculated using the median method. Each point represents the SEE for one split of the training and testing data set for each mtry and ntree combination. The residual plots are density plots of the residuals for mtry values from 1 to 9, at a constant ntree of 200.

The mean SEE varies with the number of trees (Figure 2) where the smaller number of trees performs marginally worse than the larger number of trees (Figure 2b). This is because the number of trees is a convergent parameter as seen in other studies focused on hyperparameter tuning of random forests (Oshiro et al., 2012; Probst et al., 2019; Sage et al., 2020). Figure 2 (b, e) show a slight negative trend in both the pressure and temperature between 101 and 201 trees, but we stress that the difference is marginal. Clearly, we can see that the mtry has a larger control on the performance of the model as expected from results in previous studies (Probst et al., 2019; Simm et al., 2014). As seen in Figure 2 (a, d), the larger mtry performs better (e.g., at ntree = 201 and mtry of 6 gives a mean SEE of 3.13 kbar and 70°C) than the smaller mtry (e.g., at ntree = 201 mtry of 1 give a mean SEE of 3.77 kbar and 83°C) for both the mean SEE and residuals. At mtry greater than 6, any difference is minor (±0.01 kbar), and so to limit computational cost, an mtry of 6 should be used. This is counter to the package default, which is one third the number of total variables. A similar trend is observed in the calculated IQR. However, when considering data with the inclusion of liquid—crystal pairs, the new maximum mtry is 18 and hence a new mtry needs to be considered. We performed further testing on the model with the increased maximum mtry and found that although the computational time increased, the models followed the same pattern as clinopyroxene only models whereby performance is relatively insensitive to ntree value and the mtry is optimized at about two thirds of the total variables (Figure S3 in Supporting Information S1). As such, we suggest users to select an ntree of 201 and an mtry equal to two thirds of the total input variables, which are hyperparameters that will minimize the computational cost without sacrificing accuracy (clinopyroxene oxides in the case of thermobarometry).

The package extraTrees also provides the option to vary the number of cuts at each node. This is conceptualized in a classification model for grouping people on the basis of hair color: instead of discriminating between black or blonde hair (binary choice with 1 cut), brown hair and red hair can be considered as additional options (3 cuts). While the default is 1 cut (binary; two branches at a node), increasing the number of cuts to 3–5 may yield marginal performance improvements (Simm et al., 2014). Upon further testing, we found that the additional number of cuts from 1 to 2 does minorly improve the model. However, the minor improvement of the SEE is less than 0.02 kbar and 0.5°C and so is not worth the significant increases in computational cost (Figure S4 in Supporting Information S1). Therefore, we continue to use the default of 1 cut.

### 3.2 Mean, Mode, and Median Estimates

**Figure 3:**
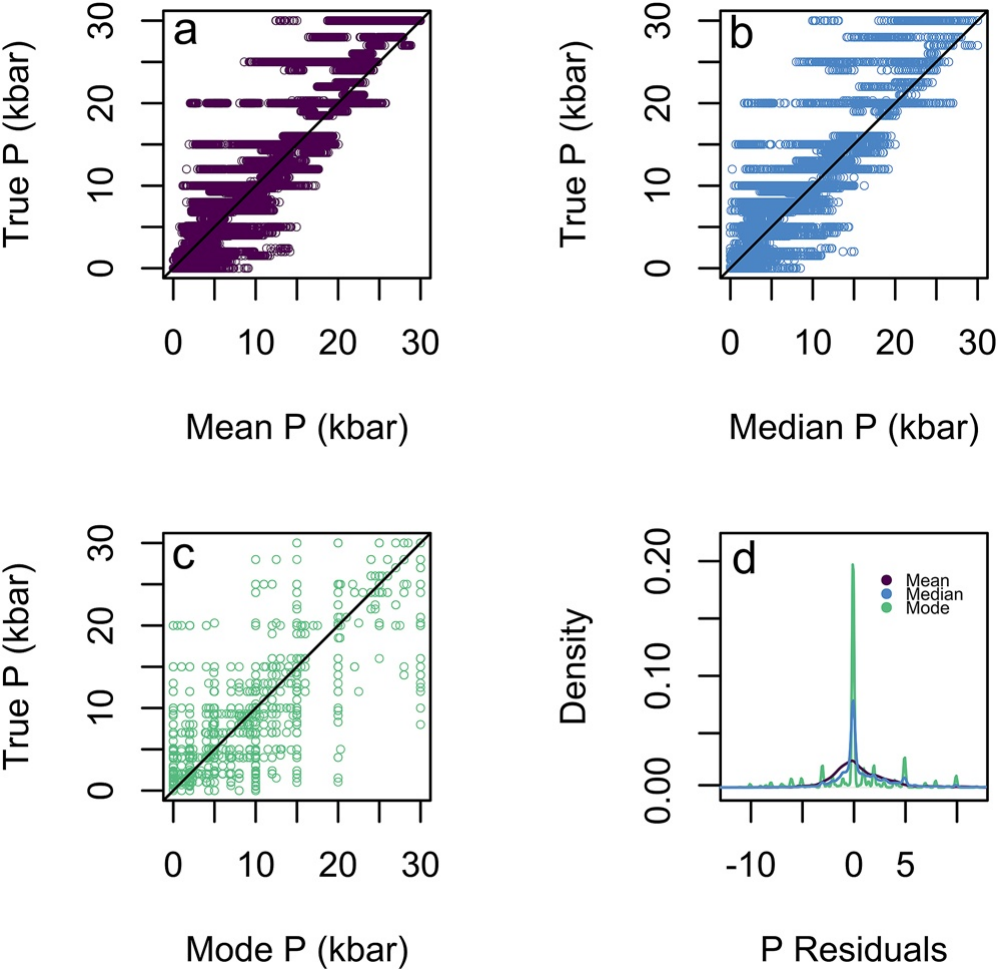
Mean (SEE = 3.20 kbar, *R* ^2^ = 0.863) (a), median (SEE = 3.3 kbar, *R* ^2^ = 0.861) (b), and modal (SEE = 4.0 kbar, *R* ^2^ = 0.782) (c) pressure determinations for the 200 test datasets versus their true pressure (44400 points plotted). (d) Density plots of the residuals for the mean, median, and mode. Here we see that the mean and median are similar in their estimates, but when the residuals are compared the median performs much better. The mode has a high concentration of points at 0 residuals but shows a many more poor residuals at higher values, thus the median is the best option to use to get the most accurate data.

As previously discussed, the random forest is composed of several hundred decision trees as defined by the user via the function argument ntree. For each input sample, ntree estimates of pressure and temperature are generated, and the final value is chosen from this voting distribution. The default option of the R package extraTrees in regression is for the forest to choose the mean of all decision tree outputs as the final pressure or temperature (Simm et al., 2014). However, the distribution of the decision trees may not be a perfect Gaussian distribution and thus we have also considered the median and modal estimates of the pressure and temperature voting distributions in addition to the mean (Figure 3).

To evaluate the performance of the mean, median, and modal estimates, we create pressure and temperature models using the entire calibration data set for clinopyroxene. Figure 3 shows estimated pressure plotted with respect to the true pressures for all 200 test data splits, using the mean, median, and modal method. The residuals, the difference between the estimated and true pressure and temperature estimates, show the widest distribution of residuals for the mean, extending out to ±5 kbar, indicating a poorly performing model. When we consider the SEE, the median outperforms the mode (median SEE = 3.3 kbar, mean SEE = 3.20 kbar, and mode SEE = 4.0 kbar). R^2^ shows the best performance for the mean (*R* ^2^ = 0.863) and median (*R* ^2^ = 0.861) both of which offer significant improvements compared to the model using a modal estimate for prediction (*R* ^2^ = 0.782).

### 3.3 Inclusion of Equilibrium Liquids

**Figure 4:**
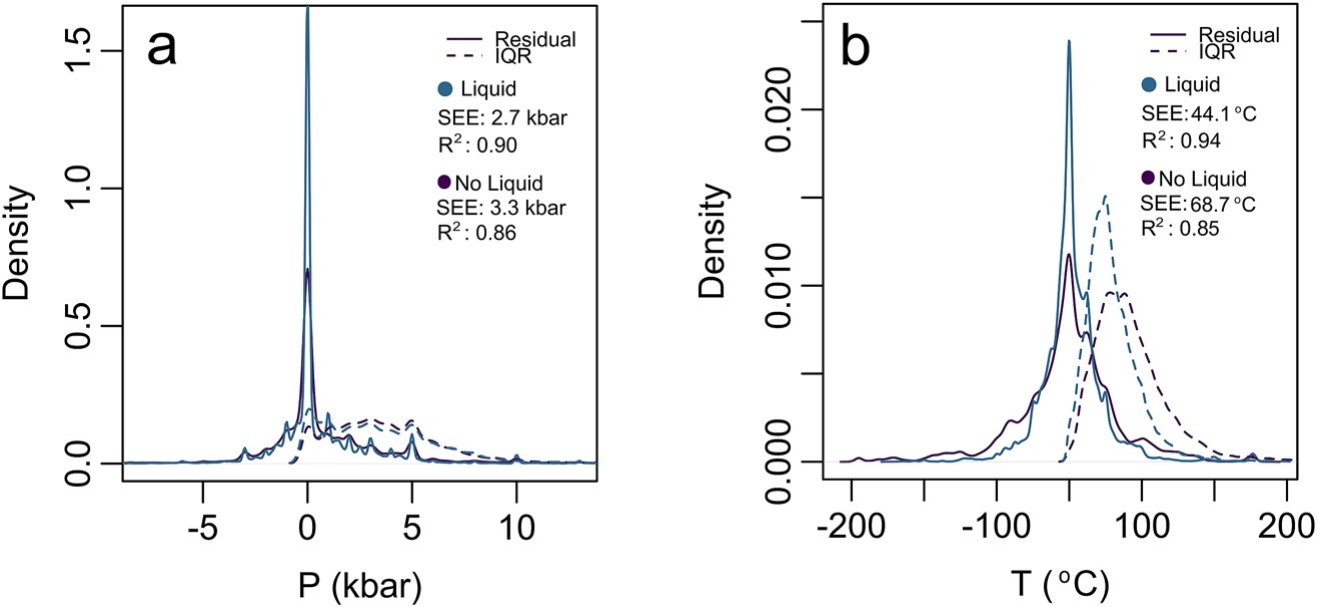
Residuals (solid) and IQR (dashed) density plots for liquid and no liquid models, plots are for pressure (a) and temperature (b).

Major element partitioning within the crystal structure of clinopyroxene is not solely sensitive to pressure and temperature but also dependent on chemical availability of elements in the residual liquid (melt). Thus, in systems for which both clinopyroxene and equilibrium melt chemistry are available, we have also calibrated a clinopyroxene‐liquid thermobarometer in addition to the clinopyroxene only model we have presented thus far. All points in the calibration data set included liquid. Performance testing of the two models (Figure 4) reveals that, as expected, the model performs more favorably when liquid data are included as this helps to isolate the pressure‐temperature dependence from the melt compositional dependence in the clinopyroxene. Figure 4 shows that liquid model curves have a higher point density at 0 for the residuals, and IQR ranges closer to 0. For pressure, the SEE decreases by 0.5 kbar and the R^2^ changes from 0.86 to 0.90. For temperature, the difference is even more striking where the SEE decreases by almost half from 68.7°C to 44.1°C and the R^2^ improves from 0.85 to 0.94.

## 4 Discussion

### 4.1 Mean, Mode, and Median: Which to Use?

Fundamentally, if the distribution of decision trees produces a perfect Gaussian distribution, then using the mean, which is the default of extraTrees, is appropriate. However, the distribution is often not a perfect Gaussian curve. Additionally, some voting distributions may have very wide uniform distributions also indicating an estimate with a low degree of certainty. Other voting distributions show sharp peaks at a given value followed by small, wide tails to low and/or high pressure/temperature. Such tails from poorly performing trees lead to overestimates of pressure or temperature due to unfair weighting by the mean of the distribution. Poorly behaving trees can result from elements being selected for decision tree nodes, which do not have a strong control on the variation of clinopyroxene unit cell parameters: these features ultimately govern the relationship between pressure, temperature, and mineral chemistry (Nimis & Ulmer, 1998).

**Figure 5:**
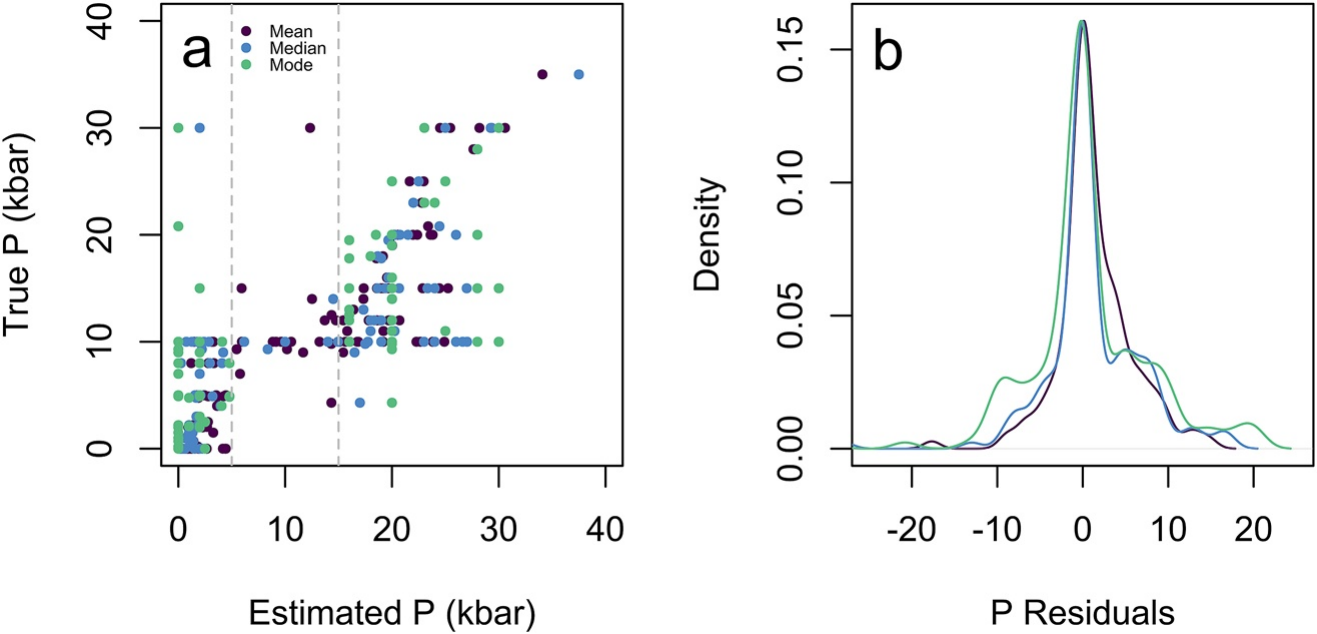
Results from a model with a pressure gap from 5 to 15 kbar forced into the calibration data set (gray dashed lines). Clearly seen in a and b is the poor performance of the modal estimates.

Mean, median, and modal models all perform well although the residuals from the modal and median model are preferable to the mean (Figure 3d). Considering the R^2^ of modal versus median model estimates, modal estimates (0.782) are lower than that of the median (0.861). Despite the modal model showing a marginally tighter distribution of residuals, it has a fundamental flaw that is shown in Figure 5. Here, 10% of the calibration data set was randomly extracted and a pressure gap between 5 and 15 kbar was forced into the training data set. When the testing set is run in this pressure gapped model, the mode cannot interpolate any points in this pressure gap. Conversely, the median and mean models can close this gap by averaging values. Of course, this is an exaggerated example, but it will indeed happen on smaller scales as experiments are often lacking in intermediate values (Hirschmann et al., 2008). Natural mineral chemistry typically shows a mixture of punctuated and continuous variability (Armienti et al., 2007; Conticelli et al., 2010). Thus, we suggest that all users adopt a median value for the PT estimates.

### 4.2 Evaluating the Estimation Uncertainty

**Figure 6:**
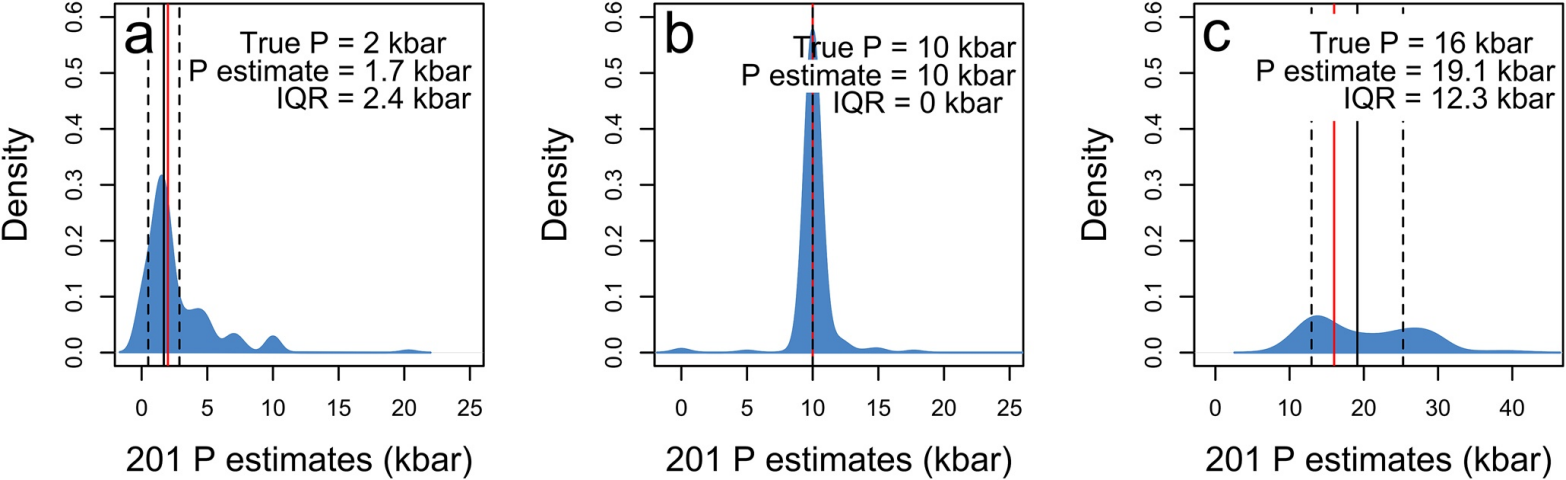
Figure explaining the components of the IQR and showing examples of samples which have generated an average (a), high (c), and low (b) IQR. Samples plotted here are the 201 estimates given from one forest for one sample. The solid black vertical line is the estimated pressure using the median method, the solid red vertical line is the true pressure, and the two black vertical dashed lines represent the IQR. Text on the plot shows the true pressure, estimated pressure and interquartile range, all in kbar.

Throughout the course of this work, we have optimized each model to give the best representation of the true (experimental) pressure and temperature. Though we have tested and optimized each model, there remain datapoints with high residuals, giving a poor estimate relative to the true experimental value (e.g., Figure 3). With natural samples, the true pressure or temperature value is unknown and if they exist in natural data sets, these anomalous samples cannot be identified. Thus far, we have assessed the overall performance of the calibrated models by using a mean SEE for each model (Figure 2). However, this averaged SEE characterizes the model's ability to predict an entire test data set and so does not provide a unique representation of the uncertainty of any specific sample. To permit closer assessment of uncertainty, we use the interquartile range (IQR) of the voting distribution (Figure 6) to assign the confidence interval of individual natural samples. The premise is that although certain individual trees may perform poorly (see Methods above), a model that performs well overall will result in a high number of trees, predicting a pressure or temperature close to the true value. This will manifest in a voting distribution that is tight, indicating that the model has a high degree of certainty in its prediction. Users are encouraged to investigate the distributions of the PT estimates, especially in the case of a bimodal distribution or a particularly skewed distribution.

To understand why some samples yield a high IQR and some low, we can examine the test and train data sets to look at some examples of significant variations in IQR. In Figure 6, we see three examples of pressure estimates, provided by the 201 trees, represented by a density curve. The solid black vertical line is the estimated pressure using the median method, the solid red vertical line is the true pressure, and the two black vertical dashed lines represent the IQR. In Figure 6a, we see a standard IQR value, where the true (2.0 kbar) and estimated (1.7 kbar) pressures are relatively close and the IQR is a reasonable value (2.4 kbar). Figure 6b shows the ideal case where the IQR is too small to see on the plot, and the estimated and true pressures are identical (10.0 kbar). Figure 6c shows an example with a large IQR (12.3 kbar) and different true (16.0 kbar) and estimated (19.1 kbar) pressures. In this final case, we see that the true pressure is still plotting within the IQR; however, we recommend users treat any data with an IQR higher than double the overall model SEE with a healthy amount of caution. Additionally, we stress that users should consider their results within a textural context to see the effect on zoning patterns (resorption, sector zoning, and disequilibrium) with respect to the PT estimates as well as the IQR.

**Figure 7:**
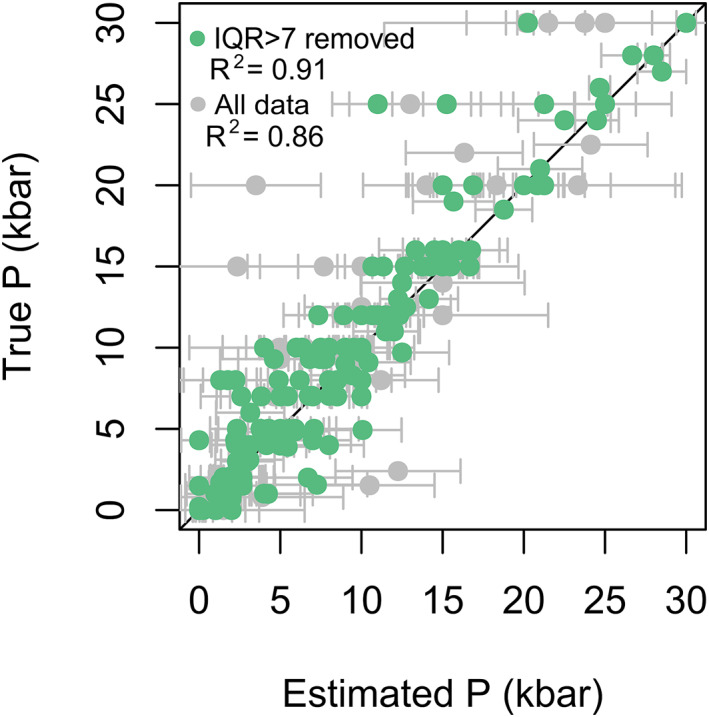
Single split of the test/train data set plotted with the IQR as one would with error bars in gray. Points in gray are all the data and in green represent the data filtered to remove any data with an IQR larger than 7 kbar.

The user may either present their natural data with the IQR as an uncertainty (e.g., error bar) or use the IQR as a metric for post‐estimate filtering. Figure 7 shows the results of a single split of the test and train data set in gray points. This plot shows IQR plotted as pseudo error bars in which almost all points within their IQR ranges lie on the 1:1 line. The datapoints in green show an example of IQR filtering, where data with an IQR larger than 7 are removed. We observe that points qualitatively identified as outliers are removed by this filtering, and the points that remain plot close to the 1:1 line. The same principle can be applied to temperature estimates. This approach encourages users to carefully consider their own data on a point‐by‐point basis to determine their contribution to the final target of the study. Analyses returning a low IQR may be considered more robust, and interpretations can be based on these points with greater confidence. This is a noteworthy and novel advantage of random forest thermobarometry with respect to other methods.

### 4.3 Pressure Filtering

Experiments that are performed under pressurized conditions require complex machinery and sometimes large time commitments (Holloway & Wood, 2012; Kägi et al., 2005; Leinenweber et al., 2012). Thus, the suite of data in the calibration data set is heavily skewed toward experiments carried out at lower pressures (≤2 kbar). This is especially true for experiments performed at 1 atm, which comprise 29% of the filtered calibration data set, likely owing to the limited range over, which pressure assemblies can effectively and safely operate at magmatic temperatures (Shaw, 2018b). We had concerns that this might unevenly skew the barometer estimates to lower pressures. To test this, we ran several models: the base model (or “mantle model”; *P* ≤ 30 kbar) and the “crustal model” (*P* ≤ 15 kbar) as chosen for the crustal range on the basis of the average crustal thickness (Kopp et al., 2011; MacKenzie et al., 2008; Tewari et al., 2018). Finally, we ran these two models with 1 atm experiments included and excluded.

**Figure 8:**
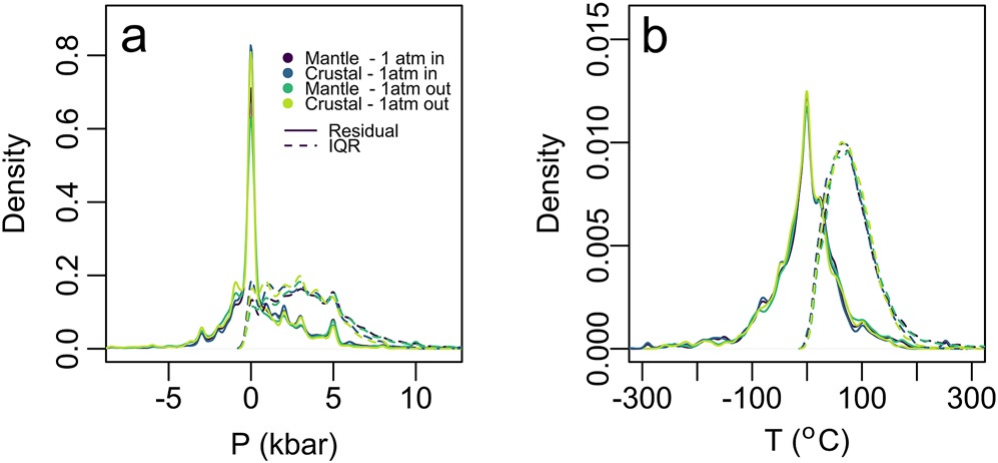
Residuals (solid) and IQR (dashed) density plots for the pressure filtered models mantle (0–30 kbar), crustal (0–15 kbar) with and without the 1 atm experiments. Plots are for pressure (a) and temperature (b).

As seen in Figure 8, there is not a strong effect on the residuals for the four models in pressure or temperature space. There is a slight effect on the IQR with the density curves of crustal models for both pressure and temperature, showing a higher density of low IQR values than the mantle model, and the density of the pressure residuals seems to be minorly denser at 0 for the crustal model (Figure 8). Considering this quantitatively, we assess the average R^2^ and SEE values over the 200 test and train data set splits. For the “mantle‐1 atm” in the model, the SEE is 3.3 kbar and 68.6°C, and R^2^ of 0.86 for pressure and 0.85 for temperature, whereas the “crustal‐1 atm in” model gives a lower SEE of 2.4 kbar and 68.6°C and but a much lower R^2^ of 0.76 for the pressure model and 0.77 for the temperature model. When we consider the 1 atm excluded models, the “mantle‐1 atm out” model gives an SEE of 3.2 and 65.0°C and an R^2^ of 0.85 for pressure and 0.87 for temperature, and the crustal model shows a similar trend of a lower SEE 2.3 kbar and 62.9°C and a worse R^2^ of 0.75 for pressure and 0.82 for temperature.

Given this information, we must also consider one of the most striking limitations of a random forest algorithm—that it cannot extrapolate data. This means that natural clinopyroxenes crystallized within the mantle, which are input into a crustal model, may yield anomalously low‐pressure estimates. Thus, even though the crustal model has shown slight advantages with respect to IQR and average SEE, we suggest that users employ the mantle model with the 1 atm experiments included. This is even more critical for compositions where experimental data are sparse. Alternatively, the “choose your own adventure” code contains instructions for tailoring models to user requirements, such as changing bounds of pressure filtering for application to areas with thicker (continental) crust (Bloch et al., 2017).

### 4.4 Adding Liquid Data to the Model

As demonstrated in Figure 4, adding equilibrium liquid data improves the model (SEE is lower by >0.5 kbar and >30°C), and so quantitatively it seems favorable to use liquid data if it is available to users. In nature, however, opportunities for reliable coexisting melt measurement may be rare. Melt inclusions have been shown to suffer from post‐entrapment crystallization, which alters their composition of the melt inclusion (Bucholz et al., 2013; Danyushevsky et al., 2002; Steele‐macinnis et al., 2011) or precipitation of daughter minerals at the edges of the melt inclusions (Moore & Carmichael, 1998; Venugopal et al., 2020). Additionally, melt inclusions may be absent in crystals or overrepresented in core or rim domains due to favorable growth along cracked surfaces (Faure & Schiano, 2005) or during heating, dissolution, and reprecipitation (Cashman & Blundy, 2013; Edmonds et al., 2016; Nakamura & Shimakita, 1998). Measuring matrix glass and crystal rim as the mineral‐liquid pair is the most common metric for clinopyroxene—liquid thermobarometry. This may generate a bias in P‐T estimates toward the final equilibration conditions of the upper part of the magmatic system. Previous works have investigated the use of melt matching algorithms to circumvent lack of liquid but others have found this to impose an additional uncertainty to the estimates so we did not explore further (Neave et al., 2019; Neave & Putirka, 2017; Petrelli et al., 2020).

By using single‐phase thermobarometers, the entire protracted history of the crystal can be measured, which can recover the full extent of crystallization P‐T in trans crustal magmatic systems (Annen et al., 2006; Christopher et al., 2015; Sparks et al., 2019). Regardless, the performance of the liquid model is clearly superior to the crystal only model, so we suggest that users of the clinopyroxene‐liquid model keep a detailed petrological record of melt inclusions including distribution in the crystal and occurrence of mineral precipitation at melt inclusion margins.

## 5 Conclusions

We have shown that machine learning is a powerful and versatile approach to thermobarometry in agreement with other studies (Higgins et al., 2022; Petrelli et al., 2020). Through detailed testing, we have determined models that have an SEE comparable to the leading clinopyroxene thermobarometers (SEE of 3.2 kbar, 47.6°C and 4.4 kbar, 76.0°C for the liquid and no liquid models, respectively, as compared to 3.4 kbar and 125°C for the alkaline only liquid‐cpx models of Masotta et al. (2013); 1.4 kbar for the mafic models of Neave and Putirka (2017); 3.1 kbar, 2.94 kbar, and 31.4°C from equation 32a, 32c, and 33, respectively, in (Putirka et al., 1996); 2.68 kbar and 93°C for the Wang et al. (2021) model). This thermobarometer can be applied to a wider range of compositions with a similar performance as existing models. Additionally, this model as has the added benefit of error analysis on individual estimates, where users can discard poorly performing estimates if they desire. Currently, no thermobarometer is accurate enough to resolve small distinct chambers within the upper or lower crust due to residuals exceeding 1–2 kbar. Our thermobarometer remains a powerful tool used in conjunction with textural data to constrain upper and lower crustal crystallization. Our extensive calibration data set means our models are highly suited for the global range of melt compositions. Additionally, when used in combination with the IQR of the voting distributions, users can further constrain accuracy of the pressure and temperature estimates and uniquely filter these values rather than relying merely on a single SEE assessment.

Hyperparameters generally make little difference to the performance of the thermobarometer. The largest effect is the value of mtry, which at low values (1 or 2) yields poor model performance (Figure 2). Instead, the largest effect on model performance is the method of output determination, that is, whether the mean, median, or mode of the voting distribution is used to recover pressure and temperature. Here, we reveal that although the mean can provide reasonable pressure and temperature estimates, natural data for which analogous experiments are sparse may yield anomalously high‐pressure predictions for low‐pressure experiments. The mode, on the other hand, gives values with the lowest residuals but struggles to reproduce data reliably in significant pressure and temperature gaps (Figure 5a). Thus, we recommend a semi‐automated approach where users filter their data using the interquartile range of the voting distribution but rely on the median value of the predicted pressure and temperature by default. This allows for consistently lower residual values when predicting experimental data.

Two sets of codes have been created, with detailed comments and instructions, for the Earth sciences community to rapidly predict intensive parameters for natural data or create more tailored models (Appendix A). The purpose of this paper is to provide a framework for use of machine learning thermobarometry in Earth Sciences for users of widely differing computing experience. We believe that our model, given the right considerations, can result in a high‐resolution study of crustal magmatic systems. This also provides a general strategy for a machine learning approach to a single phase thermobarometry, which can be applied to other minerals. Future work will focus on testing the model with chemically independent pressure and temperature estimates and show examples of how this model can be used for different melt compositions.

## Supporting information

Supporting Information S1

## Data Availability

Version 1.1 of the software Random Forest cpx‐thermobarometer is preserved at https://doi.org/10.5281/zenodo.5838122, https://zenodo.org/record/5838122#.Yd7qmv7MI2x and is available via creative commons attribution. Any minor updates to the code will be available at https://github.com/corinjorgenson/RandomForest-cpx-thermobarometer.

## Appendix A Code Distribution and Usage

Our methodology can be widely implemented within the volcanology and petrology community. We have created two versions of the models, which we are fondly calling the “Choose your own adventure” (CYOA) model and the “Plug and play” (PnP) model. Both versions are available on Github as a comprehensive R script for download at https://github.com/corinjorgenson/RandomForest-cpx-thermobarometer and archived on Zenodo at https://zenodo.org/record/5838122#.Yd7qmv7MI2x (Jorgenson et al., 2021). In this section, we will describe how to use each of the scripts. Users who are not familiar with R are directed to “YaRrr! The Pirate's Guide to R,” where Chapter 2 has instructions for installation (https://bookdown.org/ndphillips/YaRrr/installing-base-r-and-rstudio.html Phillips, (2017). Users should have at least R version 3.5 for best results. Users who prefer to use python will find in the Supporting Information S1 python functions that modify the standard implementation of the python Exrta Trees regressor.

1. Choosing the model
  The “Plug and Play” models are created using a defined set of major oxides, which a user must have in their data to use the model. The elements are SiO_2_, TiO_2_, Al_2_O_3_, Cr_2_O_3_, FeO, MgO, MnO, CaO, and Na_2_O for the clinopyroxene analysis and SiO_2_, TiO_2_, Al_2_O_3_, FeO, MgO, MnO, CaO, Na_2_O, and K_2_O for the liquid analysis. If users do not have these elements, then they must use the “Choose your own adventure” and adjust what elements are used to calibrate the model. Liquid analysis should be in equilibrium with the clinopyroxene host and the two measurements should be taken where they are in apparent equilibrium. We recommend users input their data into the.csv file “InputData,” while retaining the same column headers. If a user does not have liquid data, then they should leave columns blank or put zeros in place.
2. Choose your own adventure
  This folder comprises seven separate R scripts, which should be run in sequential order. The folder also includes the initial calibration data set as a.csv file, an example natural data set, and an R data file with oxide weights titled cpx_dat, YOUR_DATA, and OxiWeight.Rdata, respectively. A brief explanation of usage can be found in a.txt file titled README. Here, we will sequentially discuss the code for each file. We recommend between running each script, the user clears the environment and reloads the necessary files to preserve computer memory. While running this code, users should keep a keen eye on the console in case of any errors. If there are any errors, we advise clearing the environment and rerunning the code. Most of the scripts should run on the order of seconds, except for script #4. As a benchmark, running the entire CYOA cpx‐liq model on a PC (i7, 4 cores, 16GB of RAM) takes 3–5 min.
  i. Preprocessing—cpx thermobaro
    This script is used for preprocessing of the calibration data set (Table S1 in Supporting Information S1). All mineral data are recalculated according to their respective structural formula following the methodology of Deer et al. (1997). This script converts any reported Fe_2_O_3_ to FeO and normalizes the liquid data to 100 wt% anhydrous. The output is a file called raw.Rdata. You do not need to change anything in this sheet unless you change the calibration data set (e.g., to add new experimental data from the scientific literature). If the user decides to add new experiments to the calibration data set, it is imperative that they format the new data in the same way that the calibration data set is currently formatted.
  ii. Filtering—cpx thermobaro
    This script is used for filtering of the calibration data set. Our choices of chemical and P‐T filters can be found in Section 2.1. The user does not need to change anything in this script unless they desire alternative filtrations (i.e., specific compositional or pressure filters).
    Data output from script 1 (called raw) should be reloaded into the environment. This file is renamed to dat, and an extra column called Rm is added to the data frame, which will have either a Y or N, which dictates if data should be filtered (Y) or not (N).
    As outlined in Section 2.1, the Kd provides a test for equilibrium between clinopyroxene and based on the Fe/Mg ratio and can be used to filter poor quality data. Additionally, samples can be removed from the calibration data set >30 kbar where data are very sparse. Lastly, we filter for extremely low liquid SiO_2_ contents, which we have set as 35 wt.% SiO_2_ and abnormally high clinopyroxene K_2_O above 1.5 wt.%.
    The data are filtered so the samples that were assigned Y to the Rm column are removed. Finally, the calibration data set is mixed to avoid bias from the organization of the data. This filtered data frame is called input and saved as an Rdata file.
  iii. Distribute Grid Search
    The scripts Distribute Grid Search and Determine SEE are used to calculate the SEE for the final models. Two hundred test and training data sets are extracted and the model is run 200 times. This yields a distribution of SEE on which the modal SEE to be assigned to the model is calculated (see Section 2.3 for details). The number of test and train splits may be changed from the default by the user if required.
    In detail, the calibration data set is first loaded as input .Rdata. The number of test and train data sets is selected using the variable r. The test data set is ∼10% depending on how many points are present in the calibration data set (input). In the for loop (which runs r = 200 times), a grid system is defined where P/T upper/lower are the bounds for each grid square. perms provides all possible combinations for the lower P and *T* bounds and is summed with the upper bounds. sam is the grid, which is sampled in samp. One sample is selected from each of the grid squared and is added to perms. From perms, we determine the number of points in each of the grid squares and the grid squares with less than two points are removed from the sampled point (no.perms). Finally, the samples from each of the grid squares (perms) are called test.ids. The identities of the training data set are determined (those not present in test.ids) and assigned to the variable train.ids. Both the test.ids and train.ids are saved as .Rdata files.
  iv. Determine SEE—cpx thermobaro
    This code determines the average SEE for the P and T models. In this script, the user may add or remove liquid data. It is imperative that the conditions used for this script are the same as script #5. We strongly recommend you clear the environment before using this script.
    The calibration data set is loaded into the environment as input.Rdata and the test and train ids are loaded as testids.Rdata and trainids.Rdata from the previous script. Next, users can decide if they want to include liquid data in the model (liq <‐ c("Liquid")) or not (liq <‐ c("NoLiquid")). Next, elements used as features in the model are selected. The order of these elements must be the same in this script as in script #5 or the model will read the wrong elements and return a very poor prediction. Elements for the clinopyroxene are defined as the variable ox and for the liquid phase as liqox. Next, the r value (200, as in script #3) and hyperparameters are defined. We direct the reader to Section 3.1 for further information. Lastly, 1 atm experiments can be included or excluded. The calibration data set at this stage is renamed dat for the rest of the script.
    Objects id.test and id.train are used to determine the ids of the test/train sets in the dat (calibration data set) data frame. A set of empty lists are made for the data to be filled into. The for loop is run r (defaut = 200) times. For each run, the training data set is used to create the model and the test data set is input into the model from which the pressures are estimated using the median pressure determination. From this estimated pressure, the residuals, R^2^ and SEE, are calculated. This is repeated for pressure and temperature and finally loaded into output, which is reduced and saved as final.Rdata. From these 200 runs, the average SEE is determined by calculating the average SEE. This script has the longest computational time. During the run time, j is printed in the console twice (up to 200 times, once for pressure and once for temperature) to update the user on the model progress.
    The mean, median, and modal pressures are calculated. As discussed in the text, we suggest that users select the median estimate. Rerunning the script several times will incur minor differences in the SEE (∼0.2 kbar and ∼10°C). These variations are related to the randomness in the random number generator used during model generation. This effect is negligible.
  v. Final Model Training—cpx thermobaro
    This script generates the final model for natural data prediction. Once calibrated and saved, this model can be used continuously in script 6 for prediction of unknown data sets without the necessity of rerunning scripts 1–5 for the calibration data set. The models are formatted as random forest objects (P_C and T_C for the pressure and temperature models, respectively) and are saved as .Rdata files.
  vi. Filter user data—cpx thermobaro
    This script is the same as script #1 and #2 with some adjustments to avoid overwriting the calibration data set or your data. Users will need to change the code userdat <‐read.delim("InputData.txt") to reflect the title of their data or copy and paste their data into the InputData.csv file (and remove the data we have there) so that the formatting is maintained. Ensure oxides are properly suffixed (.cpx for clinopyroxene and .liq for the liquid data).
  vii. Run the model—cpx thermobaro
    This script is the final input for user data to retrieve pressure and temperature estimates. Inputted data should be filtered by script #6. The models are loaded in as P_C.Rdata and T_C.Rdata and outputted as predP and predT, respectively. Data are loaded in and subsetted for the elements used to make the final model. It is imperative that the element order is the same as chosen for model training or the outputs will be wrong.
    The code then takes the pred P and predT and calculates the respective mean, median, mode, and IQR estimates using the apply function. After the colon of each line, the data are saved as a dataframe termed OUTPUTDATA This OutputData.csv is the final file with estimated P and T values.
3. Plug and play
  This script and corresponding .Rdata files allow the user to use our predetermined models with a precalculated SEE for either liquid or no liquid data. These models are run with ntree = 201, mtry = 6 (12 for the liquid model), numcuts = 1, pressures input from 0 to 30 kbar (with 1 atm included). The SEE for the liquid model is 2.7 kbar, 44.9°C, and for the no liquid models, it is of 3.2 kbar and 72.5°C.
  This model assumes that the user has already filtered their data. Users should copy and paste their data into the example excel file InpudtData.csv and leave the column headers so the suffixes are saved. Clinopyroxene major oxides should be the same as in the model and need to be suffixed with .cpx even if using a no liquid model, and liquid/melt analysis should be suffixed with .liq. Examples and lists of the major oxides needed are listed in the script itself.
  To use the script users will need to first open R studio and comment (add a #) and uncomment (remove #) to reflect if they have liquid data or not. For example, if you are not using liquid data, then the code should appear as:
  liq <‐ “NoLiquid”
  #liq <‐ “Liquid”
  If liquid data is included the code should appear as:
  #liq <‐ “NoLiquid”
  liq <‐ “Liquid”
  After this step, the user should be able to select all the code (cmd+a for mac; ctrl+a for windows) and press run. Data are saved as a csv called OutputData.csv. The end of the script features some basic plots although we encourage users to delve into the wonderful world of plotting in R.
