# Supplementary material for: A Machine Learning‐Based Approach to Clinopyroxene Thermobarometry: Model Optimization and Distribution for Use in Earth Sciences
Source: J Geophys Res Solid Earth. 2022 Apr 9;127(4):e2021JB022904. doi: 10.1029/2021JB022904 (PMC9285709; doi:10.1029/2021JB022904)
Supplement: Supplementary file 1 — Supporting Information S1 [file JGRB-127-0-s001.docx]

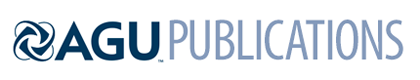


*Journal of Geophysical Research: Solid Earth*

Supporting Information for

**A machine learning based approach to clinopyroxene thermobarometry: model optimisation and distribution for use in Earth Sciences**

C. Jorgenson^1*^, O. Higgins^1^, M. Petrelli^2^, F. Bégué^1^_,_ and L. Caricchi ^1^

^1^Department of Earth Sciences, University of Geneva, Geneva, Switzerland

^2^Department of Physics and Geology, University of Perugia, Perugia, Italy

* Corin Jorgenson ([corin.jorgenson@unige.ch](mailto:corin.jorgenson@unige.ch), ORCID iD: [0000-0002-0088-6062](https://orcid.org/0000-0002-0088-6062))

**Contents of this file**

Figures S1 to S4

**Additional Supporting Information (Files uploaded separately)**

All models and model files can be found at https://zenodo.org/record/5838122#.Yd71Uv7MI2w

**Introduction**

The supporting information includes 4 figures which are supplementary to the main text.


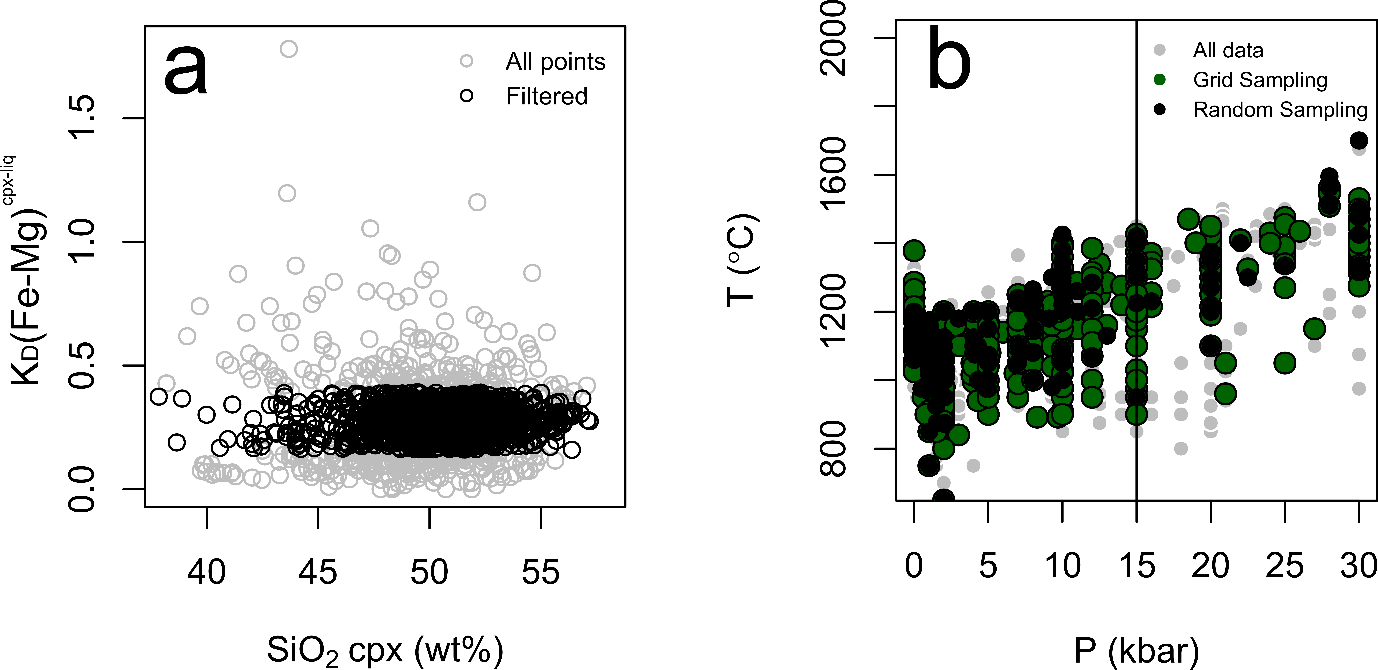


Figure S1. Data with and without K_D_(Fe-Mg)_cpx-liq_ filtration. b) pressure-temperature distribution of the calibration dataset if the test dataset is sampled with random 10% sampling and with grid sampling


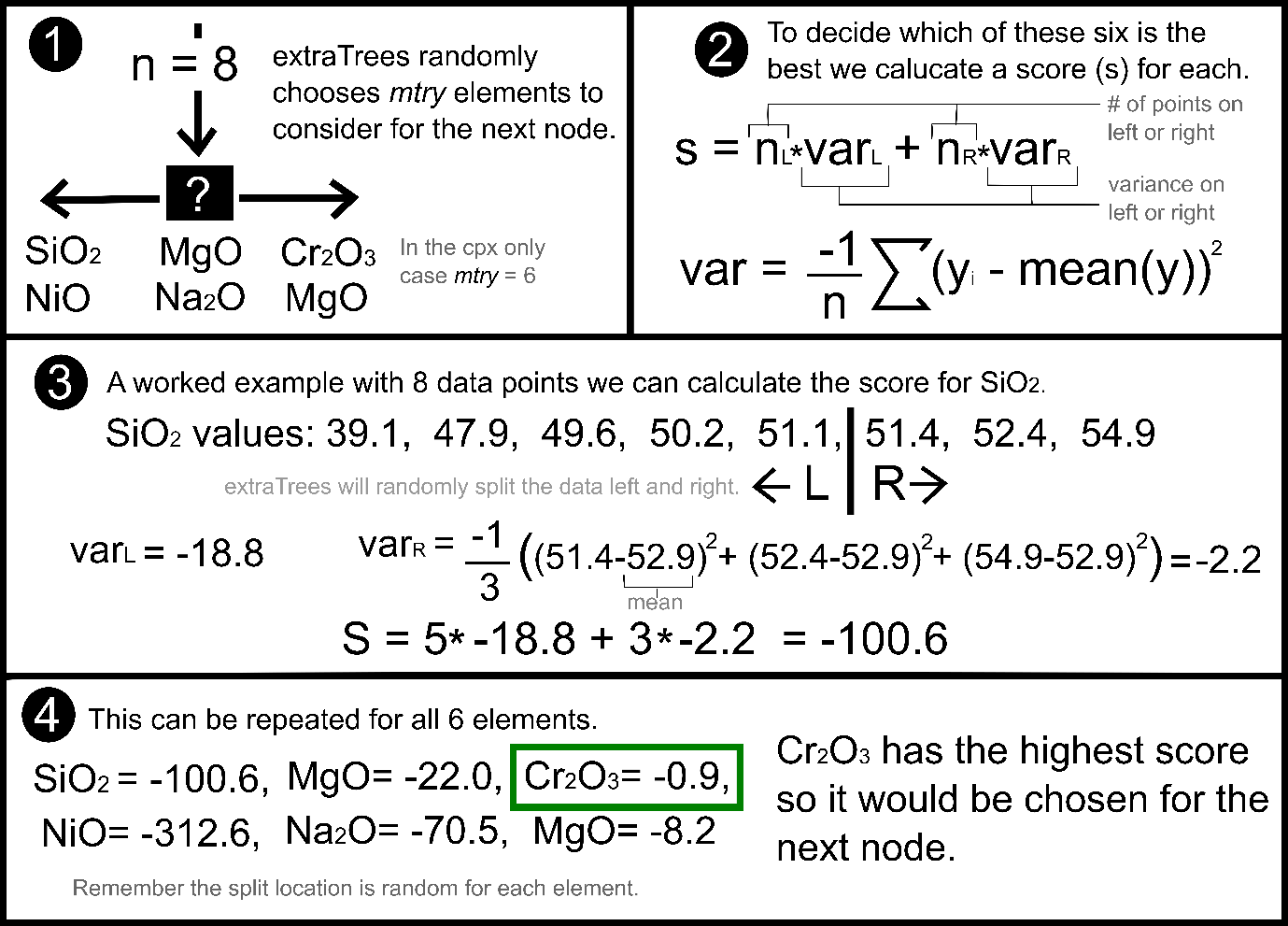


**Figure S2***.* Infographic to further explain how each node is chosen when the model constructs a decision tree. 1) Shows mtry (6) elements randomly chosen by the model, 2) Shows the equations used to calculate the score and variance for each element, 3) A calculated example of the variance and score for the right side of the split using SiO_2_ contents of clinopyroxene as an example 4) The score of each element is shown and the element with the highest variance is chosen as the node for the next split.

*
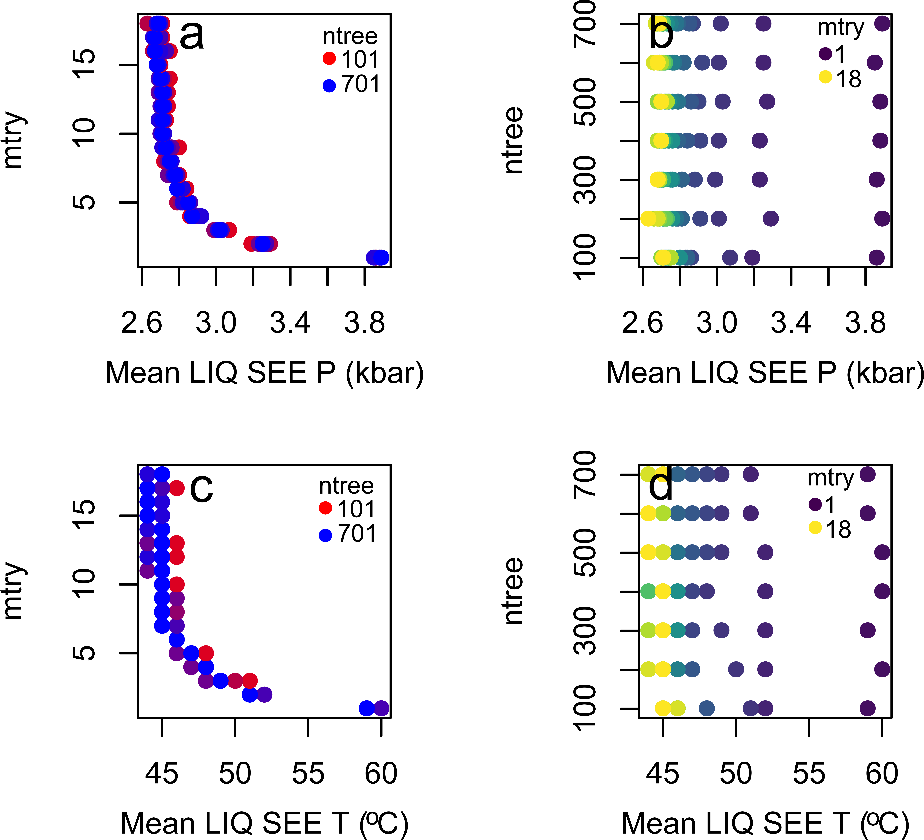
*

**Figure S3.** Variation of mtry on the SEE for P and T for the liquid data included, and thus a mtry maximum of 18 variables. Note that like the no liquid model, the performance of the liquid model seems to marginally improve at mtry > two-thirds of the variables. The number of trees has a very slight influence on SEE.


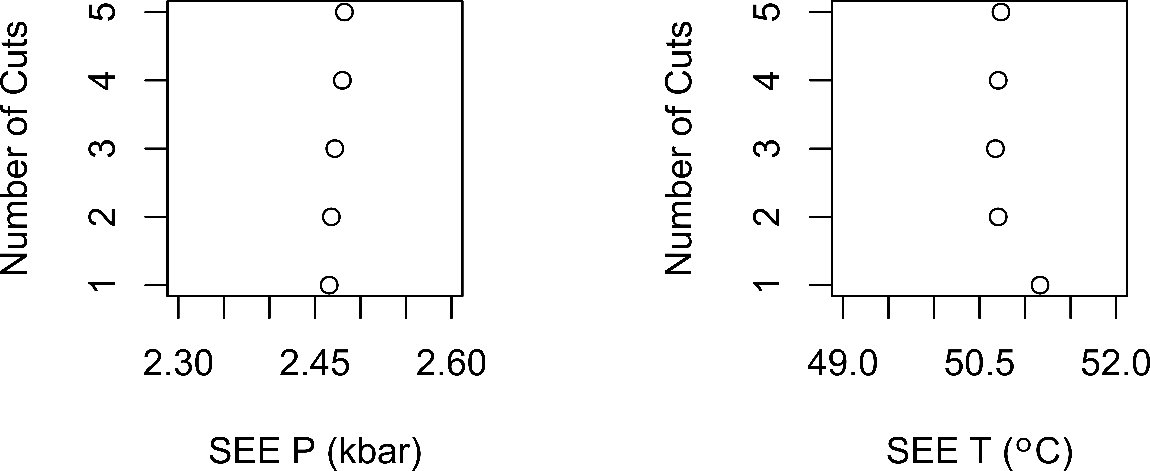


**Figure S4**. As noted in the extraTrees vignette optimization may occur when using numRandomCuts between 3 – 5. We tested numRandomCuts between 1-5 and found very little difference in performance. Thus, we decided to use numCuts = 1 to save on computational time.
